# Socio-demographic variation in adherence to the World Cancer Research Fund (WCRF)/American Institute for Cancer Research (AICR) Cancer Prevention Recommendations within the UK Biobank prospective cohort study

**DOI:** 10.1093/pubmed/fdad218

**Published:** 2023-11-20

**Authors:** Fiona C Malcomson, Solange Parra-Soto, Liya Lu, Frederick Ho, Carlos Celis-Morales, Linda Sharp, John C Mathers

**Affiliations:** Faculty of Medical Sciences, Human Nutrition & Exercise Research Centre, Centre for Healthier Lives, Population Health Sciences Institute, Newcastle University, Newcastle upon Tyne, NE2 4HH, UK; Faculty of Medical Sciences, Centre for Cancer, Population Health Sciences Institute, Newcastle University, Newcastle upon Tyne, NE2 4HH, UK; College of Medical, Veterinary and Life Sciences, School of Health and Wellbeing, University of Glasgow, Glasgow, G12 8TA, UK; College of Medical, Veterinary and Life Sciences, School of Cardiovascular and Medical Sciences, University of Glasgow, Glasgow, G12 8TA, UK; Department of Nutrition and Public Health, Universidad del Bío-Bío, Chillan, Chile; Faculty of Medical Sciences, Centre for Cancer, Population Health Sciences Institute, Newcastle University, Newcastle upon Tyne, NE2 4HH, UK; College of Medical, Veterinary and Life Sciences, School of Cardiovascular and Medical Sciences, University of Glasgow, Glasgow, G12 8TA, UK; College of Medical, Veterinary and Life Sciences, School of Health and Wellbeing, University of Glasgow, Glasgow, G12 8TA, UK; Human Performance Lab, Education, Physical Activity and Health Research Unit, University Católica del Maule, Talca, Chile; Faculty of Medical Sciences, Centre for Cancer, Population Health Sciences Institute, Newcastle University, Newcastle upon Tyne, NE2 4HH, UK; Faculty of Medical Sciences, Human Nutrition & Exercise Research Centre, Centre for Healthier Lives, Population Health Sciences Institute, Newcastle University, Newcastle upon Tyne, NE2 4HH, UK

**Keywords:** Cancer Prevention Recommendations, cancer risk, lifestyle, UK Biobank

## Abstract

**Background:**

The 2018 (WCRF)/American Institute for Cancer Research (AICR) Cancer Prevention Recommendations are evidence-based lifestyle recommendations which aim to reduce the risk of cancer worldwide. Sociodemographic factors modulate lifestyle behaviours, and both cancer incidence and survival are socio-economically patterned. We investigated adherence to these recommendations and examined patterns of adherence across sociodemographic subgroups in the UK Biobank cohort.

**Methods:**

We included 158 415 UK Biobank participants (mean age 56 years, 53% female). Total adherence scores were derived from dietary, physical activity and anthropometric data using the 2018 WCRF/AICR standardized scoring system. One-Way analysis of variance (ANOVA) was used to test for differences in total scores and in values for individual score components according to sociodemographic factors and Pearson’s *Χ*2 test to investigate associations between sociodemographic factors according to tertiles of adherence score.

**Results:**

Mean total adherence score was 3.85 points (SD 1.05, range 0–7 points). Higher total scores were observed in females, and older (>57 years), Chinese or South Asian, and more educated participants. We found significant variations in adherence to individual recommendations by sociodemographic factors including education, Townsend deprivation index and ethnicity.

**Conclusions:**

Identifying and understanding lifestyle and dietary patterns according to sociodemographic factors could help to guide public health strategies for the prevention of cancers and other non-communicable diseases.

## Introduction

Unhealthier lifestyle patterns including poor quality diets, physical inactivity and living with obesity increase risk of non-communicable diseases, such as cancer, as well as mortality.^1–3^ The Global Burden of Diseases, Injuries and Risk Factors Study 2019 estimated that environmental, behavioural and occupational risk factors e.g. alcohol and a high body mass index (BMI), accounted for 44% of cancer deaths.^3^ However, the risk-attributable cancer burden varied by geographical region and by country-level socio-demographic index.^3^ Global risk-attributable cancer deaths related to these risk factors increased by > 20% between 2010 and 2019^3^ and, in low and middle socio-demographic index countries, there has been a substantial increase in the burden of cancers associated with high BMI.^3^ Further, recent work suggests that the rising incidence of early onset (<50 years at diagnosis) cancers internationally is linked with changing patterns of lifestyle and risk factor exposures including diet and obesity.^4^ These findings emphasize the growing impact of modifiable risk factors and of sociodemographic factors on cancer incidence and cancer deaths worldwide.

Lifestyle factors are associated with risk of at least 16 cancers, and the same factors may influence cancer survival.^5^ The World Cancer Research Fund (WCRF)/American Institute for Cancer Research (AICR) Cancer Prevention Recommendations are a set of 10 lifestyle-based recommendations.^5^ A systematic review and meta-analysis found that greater adherence to the 2007 Cancer Prevention Recommendations was associated with reduced risk of breast, colorectal and lung cancer, and cancer-specific and overall mortality.^6^ However, there was significant methodological heterogeneity across studies, including differences in the approaches used to assess adherence to the recommendations. In 2019, a standardized scoring system for assessing adherence to the 2018 Cancer Prevention Recommendations, known as the ‘2018 WCRF/AICR score’, was created to facilitate comparability across studies,^7^^,^^8^ and includes seven of the Recommendations, with an optional eighth component (breastfeeding) (Supplementary Table S1). This standardized scoring system enables the assessment of levels and patterns of adherence to the Cancer Prevention Recommendations across populations, and within population subgroups that is valuable in informing development and targeting of cancer control initiatives or public health campaigns to reduce risk of other common non-communicable diseases (NCDs). Several studies from mainland Europe and the USA^9–12^ have operationalized the standardized scoring system but no studies have fully operationalized this in the UK.^13^

The age-standardized cancer incidence rate in the UK is in the upper third of rates across Europe,^14^ and survival from many cancers is lower than in several comparable high-income countries.^15^^,^^16^ Within the UK, both cancer incidence and survival are socio-economically patterned, with incidence higher and survival lower in those residing in the most deprived areas.^17^^,^^18^ Similar patterns are also seen when other markers of socio-economic position are considered, such as education, and there are sociodemographic disparities in uptake of cancer screening programmes.^19^^,^^20^ There is also evidence that cancer-related lifestyle behaviours vary by socioeconomic status. In England, smoking prevalence is higher among those who do not own a home, with lower incomes and with fewer educational qualifications.^21^ Bennett and colleagues found that > 50% of UK participants did not adhere to national dietary recommendations for carbohydrate, sugar, fibre, saturated fat and polyunsaturated fat.^22^ Female participants were more likely to exceed recommended intakes of total sugar, total fat and saturated fat and to eat below the recommended intake of dietary fibre, and male participants were less likely to meet the recommended intakes of carbohydrates, protein and polyunsaturated fat.^22^ However, that study investigated selected nutrients only and, given the focus on food-based dietary guidelines in most public health policies, including in the UK,^23^ there is a need to consider overall diet quality and dietary patterns.

Identifying lifestyle and dietary patterns according to sociodemographic factors could help guide public health strategies for the prevention of lifestyle-related cancers and other NCDs. Furthermore, identifying components of the 2018 WCRF/AICR score for which there is low adherence could guide public health strategies for cancer prevention employing the principle of proportionate universalism (i.e. resourcing and delivering of universal services at a scale and intensity proportionate to the degree of need).^24^ We hypothesized that adherence to the Cancer Prevention Recommendations (adherence score) would be patterned sociodemographically (for example, we anticipated lower adherence scores among those experiencing greater deprivation). The CALIPER UK Study is the first to fully operationalize the standardized scoring system to assess adherence to the 2018 Cancer Prevention Recommendations in the UK, using data from the UK Biobank.^25^ This paper aims to: (i) describe levels of adherence (total scores) and (ii) examine patterns of adherence across subgroups of the population within the UK Biobank.

## Methods

### Study participants

We used cross-sectional data from the UK Biobank prospective cohort study, that recruited 502 536 participants aged 37–73 years between 2006 and 2010 across 22 centres in the UK. Eligibility criteria and recruitment and follow-up methods are reported elsewhere.^26^ At the baseline study visit, a touchscreen questionnaire was used to collect data on participant characteristics including sociodemographic factors, diet, physical activity and general health; anthropometric measurements and blood samples were collected. Participants recruited between 2009 and September 2010 also completed a 24-hour dietary assessment, the Oxford WebQ^27^ and those who had provided a valid email address were invited to complete the 24-hour dietary assessment during four cycles between February 2011 and June 2012.^28^ Townsend deprivation index at recruitment was derived from the postcode of residence. Smoking status was self-reported and categorized as ‘never’, ‘previous’ or ‘current’ smoker. Educational qualifications were self-reported (International Standard Classification for Education (ISCED) for years of education equivalents are described in Supplementary Table S3). Ethnicity was self-reported.

The UK Biobank study was conducted according to the Declaration of Helsinki and ethical approval was granted by the North West Multi-Centre Research Ethics Committee (reference: 06/MRE08/65). All participants provided informed consent. The protocol for the UK Biobank study can be found at: http://www.ukbiobank.ac.uk/wp-content/uploads/2011/11/UK-Biobank-Protocol.pdf.

### Operationalization of a standardized scoring system to assess adherence to the WCRF/AICR Cancer Prevention Recommendations

We assessed adherence to the Cancer Prevention Recommendations using the ‘2018 WCRF/AICR Score’^7^ (Supplementary Table S2). Details of the UK Biobank data used, and methodology applied to derive adherence scores, are described in Supplementary Material and in Malcomson *et al..*^25^ Participants were allocated 1 point for fully meeting, 0.5 points for partially meeting or 0 points for not meeting each recommendation (component of the score). Scores for individual components were summed to yield a single score for each individual ranging from 0 to 7 points.

### Statistical analyses

Baseline continuous data were summarized as means and standard deviations (SD) and categorical data as number and percentage of participants. Participants were grouped into ‘younger’ (≤57 years) and ‘older’ participants (>57 years) by dichotomizing at the median. Participants were categorized by socioeconomic status according to tertiles of the Townsend deprivation index^29^: lowest (≤ −3.295), middle (−3.294—−1.01) and highest (> −1.01). Cohen’s *d* was used to assess the magnitude of any differences in the mean age and Townsend deprivation Index between UK Biobank participants for whom we derived a ‘total (adherence) score’ (*n* = 158 415) and participants for whom we did not have sufficient data to derive a total score (*n* = 344 121).

Differences in mean total scores according to sex and age group were analysed using two-sample t-tests and for ethnicity, Townsend deprivation index, and education using one-way ANOVA. We also analysed sociodemographic characteristics by adherence score tertiles as tertiles provide information about the distribution of adherence scores. Differences in age between tertiles were analysed using one-way ANOVA and associations between adherence tertile and sex, ethnicity, Townsend deprivation index tertile, and education using Pearson’s chi^2^ test.

Differences in values for score components and sub-components (e.g. dietary fibre intake in g/day) according to categories of socio-demographic factors and to total score tertiles were analysed using one-way ANOVA and Tukey’s post-hoc test. Pearson’s rho was used to investigate correlations between scores for individual score components. Statistical analyses were conducted using StataMP 16 (Stata Corp, Texas) and *P* values < 0.05 were considered statistically significant.

## Results

### Characteristics of UK Biobank Participants

We derived a total adherence score for 158 415 UK Biobank participants (Table 1), mean age 56 years (age range 39–72) of whom 53% were female. Most participants were White (96%). Over half of the participants reported never smoking (57%) and approximately half were educated to college or university degree (49%). The distributions of sex and ethnicity and smoking status did not differ between the 158 415 participants included in the current analysis and the 344 121 for whom it was not possible to compute a score (Supplementary Table S4). Participants in the current analysis were slightly younger (56.1 versus 56.7, Cohen’s *d* 0.08), less deprived than the rest of the UK Biobank cohort (mean Townsend deprivation index −1.63 versus −1.14, Cohen’s *d* 0.160), and more had a college or University degree (49% versus 32%).

**Table 1:** Sociodemographic characteristics of UK Biobank participants with a total adherence score and included in the present analysis

	Participants with a total score included in the present analysis
N	158 415
**Sex**	
Females (%)	84 463 (53.3)
Males (%)	73 952 (46.7)
**Age (years)**	56.1 (8.0)
**Townsend deprivation index**	−1.63 (2.8)
**Ethnicity (%)**	
White	151 646 (95.7)
Mixed	1988 (1.3)
South Asian	2175 (1.4)
Black	1743 (1.1)
Chinese	433 (0.3)
**Smoking (%)**	
Never	90 365 (57.0)
Former	56 092 (35.4)
Current	11 691 (7.4)
**Education (%)**	
College or university degree	77 903 (49.2)
A levels/AS levels or equivalent	20 832 (13.2)
O levels/GCSEs or equivalent	32 047 (20.2)
SEs or equivalent/NVQ or HND or HNC	14 896 (9.4)

Data are presented as means and standard deviation (SD) or ‘n’ and percentage (%).

Data missing: 0.1% Townsend Deprivation Index, 0.3% ethnicity, 0.2% smoking status, 8% education.

A levels: Advanced levels, AS levels: Advance Subsidiary levels, CSE: Certificate of Secondary Education, GCSE: General Certificate of Secondary Education, HNC: Higher National Certificate, HND: Higher National Diploma, NVQ: National Vocational Qualifications, O levels: General Certificate of Education Ordinary Level

### Total adherence score according to sociodemographic factors

The mean total adherence score for all participants was 3.85 (SD 1.05), was higher for female participants than for males (4.07 (SD 1.02) versus 3.59 (1.01)), and for older (>57 years) compared with younger participants (3.87 (SD 1.03) versus 3.83 (1.07)) (Table 2). Total scores for ethnic subgroups differed significantly from each other, except for White versus Black participants (*P* = 0.707). Chinese participants had the highest total score (4.49 (SD 0.91)) while that for Whites was lowest (3.84 (SD 1.04)). Total score did not differ by Townsend deprivation index but differed by educational attainment—mean score for those with college or university degree was 3.96 (SD 1.03)) compared with 3.65 (SD 1.04) for those with the lowest educational attainment.

**Table 2:** Total adherence scores by sociodemographic factors

	Total score, mean (SD)	*P*-value
**All participants**	3.85 (1.05)	
**Sex**		**<0.001** ^a^
Females	4.07 (1.02)	
Males	3.59 (1.01)	
**Age**		**<0.001** ^a^
Younger (≤57 years)	3.83 (1.07)	
Older (>57 years)	3.87 (1.03)	
**Townsend deprivation index tertile**		0.135^b^
Lowest (≤ −3.295)	3.86 (1.02)	
Middle (−3.294 – −1.01)	3.84 (1.10)	
Highest (> −1.01)	3.85 (1.10)	
**Ethnicity (%)**		**<0.001** ^b^
White	3.84 (1.04)	
Mixed including others	4.08 (1.07)	
South Asian	4.21 (1.04)	
Black	3.87 (1.09)	
Chinese	4.49 (0.91)	
**Education (%)**		**<0.001** ^b^
College or university degree	3.96 (1.03)	
A levels/AS levels or equivalent	3.83 (1.05)	
O levels/GCSEs or equivalent	3.75 (1.06)	
SEs or equivalent/NVQ or HND or HNC	3.65 (1.04)	

A levels: Advanced levels, AS levels: Advance Subsidiary levels, CSE: Certificate of Secondary Education, GCSE: General Certificate of Secondary Education, HNC: Higher National Certificate, HND: Higher National Diploma, NVQ: National Vocational Qualifications, O levels: General Certificate of Education Ordinary Level

^a^Differences tested using two-sample T test.

^b^Differences tested using one-way ANOVA.

### Participant socio-demographics according to score tertiles

Table 3 describes the sociodemographic characteristics of participants by tertile of adherence score. Males were predominant in the lowest tertile (57.6 versus 42.4% for males and females, respectively) but made up a much smaller proportion of those in the highest tertile of adherence score (33.4 versus 66.6% for males and females respectively) (*P* < 0.001). The proportion of White participants decreased with increasing score tertile (from 96.6 in the lowest to 94.6% in the highest tertile) with increasing proportions of Mixed, South Asian and Chinese participants with increasing score tertile (*P*< 0.001). The proportion of participants with a college or university degree rose from 48.7% (lowest tertile) to 58.7% in the highest score tertile (*P* < 0.001).

**Table 3:** Sociodemographic characteristics of included participants according to tertiles of Adherence Score

Total Adherence Score Tertile	Lowest (0–3.5)	Medium (3.75–4.25)	Highest (4.5–7)	*P* value
**N**	64 766	43 096	50 553	
**Sex**				**<0.001**
Females (%)	27 435 (42.4)	23 357 (54.2)	33 671 (66.6)	
Males (%)	37 331 (57.6)	19 739 (45.8)	16 882 (33.4)	
**Age (years)**	55.9 (8.0)	56.3 (8.0)	56.2 (7.9)	**<0.001**
**Townsend deprivation index**				**<0.001**
Lowest (≤ −3.295)	21 274 (32.9)	14 775 (34.3)	16 701 (33.0)	
Middle (−3.294 – −1.01)	21 594 (33.3)	14 527 (33.7)	16 621 (32.9)	
Highest (> −1.01)	21 811 (33.7)	13 755 (31.9)	17 174 (34.0)	
**Ethnicity (%)**				**<0.001**
White	62 532 (96.6)	41 309 (95.9)	47 805 (94.6)	
Mixed	661 (1.0)	530 (1.2)	797 (1.6)	
South Asian	606 (0.9)	582 (1.4)	987 (2.0)	
Black	711 (1.1)	452 (1.1)	580 (1.2)	
Chinese	77 (0.1)	111 (0.3)	245 (0.5)	
**Education (%)**				**<0.001**
College or university degree	28 648 (48.7)	21 530 (54.3)	27 725 (58.7)	
A levels/AS levels or equivalent	8630 (14.7)	5742 (14.5)	6460 (13.7)	
O levels/GCSEs or equivalent	14 342 (24.4)	8446 (21.3)	9259 (19.6)	
SEs or equivalent/NVQ or HND or HNC	7202 (12.2)	3922 (9.9)	3772 (8.0)	

Data are presented as number of participants (n) and percentage (%) in parentheses, unless otherwise stated. Pearson’s chi^2^ test was used to test for differences in sex, ethnicity, Townsend deprivation index tertile, and education according to tertiles of Adherence Score.

Data for age are presented as means (SD) and analysed using Two-sample T-test.

Data missing: 0.1% Townsend Deprivation Index, 0.3% ethnicity, 8% education.

A levels: Advanced levels, AS levels: Advance Subsidiary levels, CSE: Certificate of Secondary Education, GCSE: General Certificate of Secondary Education, HNC: Higher National Certificate, HND: Higher National Diploma, NVQ: National Vocational Qualifications, O levels: General Certificate of Education Ordinary Level

### Adherence to individual components of the score

**Fig. 1:**
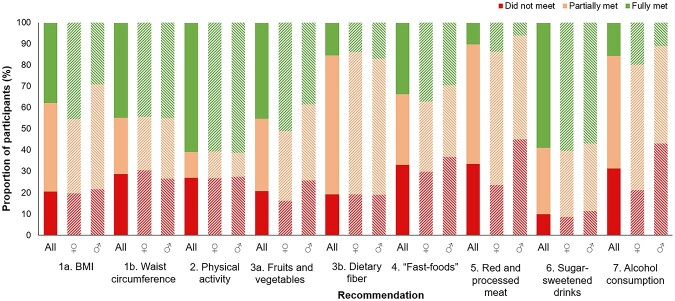
Adherence to individual components and sub-components of the score according to sex. Bars represent data for all participants and for females and males separately. The recommendation ‘1. Healthy weight’ is divided into two sub-components: (a) BMI and (b) waist circumference. The recommendation ‘3. Wholegrains, vegetables, fruit, and beans’ is divided into two sub-components: (a) fruit and vegetables intake and (b) dietary fibre intake. BMI: body mass index

Figure 1 illustrates the proportion of participants not meeting (0 points), partially meeting (0.5 points, or 0.25 for sub-components) or fully meeting (1 point, or 0.5 points for sub-components) each component of the score. Adherence was highest for the ‘physical activity’ recommendation (61% fully adhered), and the ‘sugar-sweetened drinks’ recommendation (59% fully adhered). Less than half (45%) of the participants fully adhered to the fruits and vegetables sub-component, with substantially more females than males adhering to this recommendation (51% versus 38%, respectively). For the ‘healthy weight’, recommendation, 38% and 45% of participants met the sub-recommendations for BMI and waist circumference, respectively.

Approximately one third of participants did not adhere to the ‘red and processed meat’ or ‘alcohol consumption’ recommendations. For both recommendations, the proportion not meeting the recommendation was around twice as high in males as in females (red and processed meats: 45% versus 24%; alcohol consumption: 43% versus 21%). Only 15% of participants met the recommended intake for dietary fibre (Fig. 1).

Values for score components according to tertiles of total score are summarized in Supplementary Table S5. Intakes of fruit and vegetables and of dietary fibre were lowest in participants in the lowest tertile of total score (302 g/day (SD 212) and 19.6 g/day (SD) for fruit and vegetables and dietary fibre, respectively) and greatest for those in tertile 3 (546 g/day (279) and 25.1 g/day (9.1) for fruit and vegetables and dietary fibre, respectively) (*P* < 0.001). In contrast, BMI and waist circumferences, as well as the intakes of red and processed meat, sugar-sweetened drinks, alcohol consumption and the proportion of total energy from [adapted] ultra-processed foods were significantly lower in the higher two score tertiles (*P* < 0.001). Mean BMI of participants in the highest score tertile was 24.7 kg/m^2^ (SD 3.5), compared with 28.7 kg/m^2^ (SD 4.8) in the lowest score group. Participants in the lowest score grouped consumed, on average, 66% of their total energy intake from ultra-processed foods, compared with 40% in the highest score tertile. Intake of red meat was 40% higher and of processed meat was over 2-fold greater in participants in the lowest compared with the highest score tertile. The mean intake of sugar-sweetened drinks in participants in the lowest score tertile was 3-fold greater, and of alcoholic drinks was 50% higher, in participants in the lowest score tertile compared with those in the highest score tertile.

### Values for components of the score according to sociodemographic factors

**Fig. 2:**
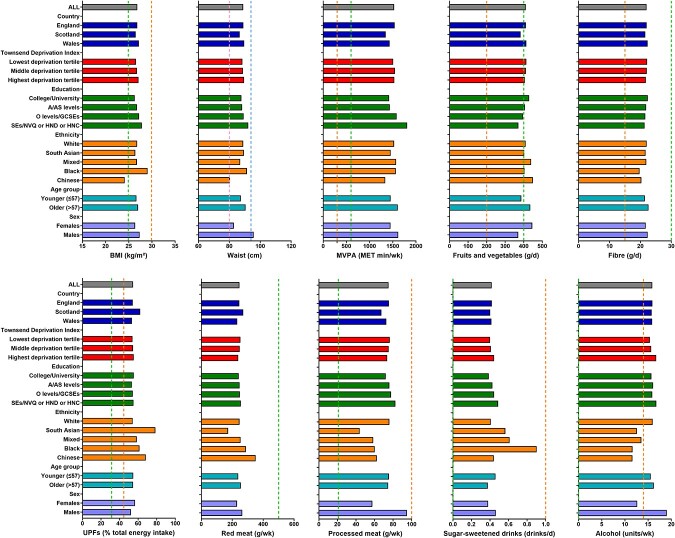
Mean values for score components overall and by sociodemographic factors. Data are presented as means. The dotted lines represent cut-offs for full and partial adherence to the score component. For waist circumference, the dotted lines represent the cut-ofs for full adherence for female and fpr male participants. For waist circumference, the pink and blue dotted lines represent the cut-offs for full adherence for female and male participants, respectively. BMI: body mass index. MVPA: moderate-vigorous physical activity. UPFs: ultra-processed foods.

The mean BMI of participants was 26.8 kg/m^2^ (Fig. 2) and was above the cut-off of 24.9 kg/m^2^ for all ethnic groups except the Chinese participants (mean BMI 24.2 kg/m^2^), who also had a mean waist circumference (88 (SD 9.3) and 76 (SD 8.7) cm for men and women, respectively) below the relevant cut-offs (94 and 80 cm for men and women, respectively). BMI and waist circumference differed significantly between all subgroups for all sociodemographic factors with the exception of White versus South Asian and White versus Mixed (BMI only) (*P* < 0.001). Physical activity differed significantly between all subgroups for all sociodemographic factors (*P* < 0.001), except for participants educated to College/University degree versus A/AS levels.

Participants recruited from Scotland, those with lower educational attainment, younger participants (≤57 years) and males reported mean intakes of fruits and vegetables <400 g/day, the cut-off for this sub-component (*P* < 0.001). For all sociodemographic subgroups, the mean intake of dietary fibre was below the recommended intake (30 g/day). Fruit and vegetable and dietary fibre intake differed (*P* < 0.001) between subgroups for all sociodemographic factors except for ethnic groups, and Townsend deprivation index where intakes were lower in the most deprived groups compared with other tertiles.

On average, participants obtained 55% of total energy intake from ultra-processed foods. Participants from Scotland (mean 62% energy from ultra-processed foods, females (57%) and South Asian participants (79%) consumed more ultra-processed foods. The mean red meat intake for each sociodemographic subgroup was below the cut-off of 500 g per week to fully adhere to this score component. In contrast, mean intake of processed meat was above the cut-off of 21 g per week for all sociodemographic subgroups. Red and processed meat intake varied significantly by subgroup for all sociodemographic factors (*P* < 0.001), except there were no differences in red meat intake between participants with A/AS Levels versus O Levels/GCSEs and in both red and processed meat intake between some ethnic groups. For example, younger participants (≤57 years old) consumed slightly more red and processed meat (167 and 73 g/day, respectively), compared with older participants (164 and 71 g/day, respectively). Participants recruited in Scotland consumed the highest amounts of red meat (178 g/day) but the lowest amounts of processed meats (68/g/day). Male participants consumed more red and processed meats (171 and 79 g/day, respectively) compared with females (159 and 61 g/day, respectively). Consumption of sugar-sweetened drinks and of alcohol differed significantly between groups for all sociodemographic factors except recruitment country. On average, participants drank ~16 units of alcohol per week (19 for males and 13 for females).

### Correlations between adherence to individual components of the score

**Fig. 3:**
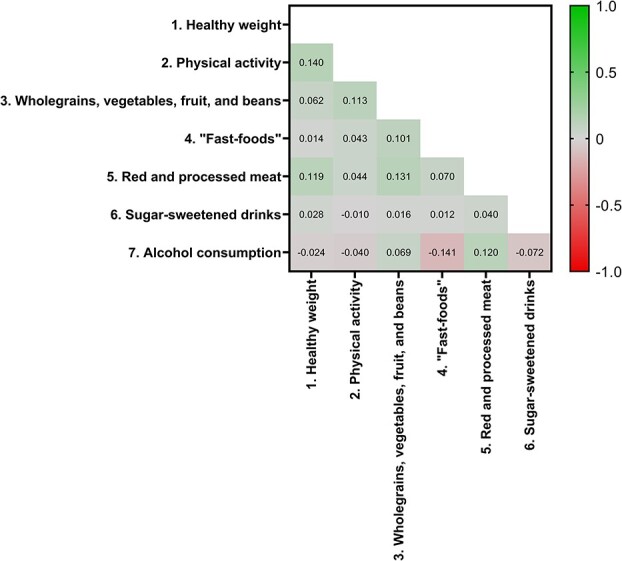
Heatmap showing correlations between adherence scores for individual components of the score^1^. A higher score represents greater adherence to the recommendation (i.e. greater levels of physical activity or lower intake of sugar-sweetened drinks).^1^Data are presented as Pearson’s correlation coefficient. All correlations were statistically significant (*P*< 0.001).

There were positive correlations between scores for: (i) the body weight and physical activity recommendations, (ii) the fruits, vegetables and dietary fibre recommendation and physical activity, ultra-processed food intake, and red and processed meat intake and (iii) the red and processed meat recommendation and body weight and alcohol intake (Fig. 3). In contrast, there were inverse correlations between scores for the recommendation to limit alcohol intake and ultra-processed food consumption, sugar-sweetened drinks intake and physical activity.

## Discussion

### Main finding of this study

Our study is the first to assess comprehensively overall adherence and socio-demographic variations in adherence to the 2018 Cancer Prevention Recommendations in a UK population. We found significant variations in total adherence scores and in adherence to individual components by sociodemographic factors including education, Townsend deprivation index and ethnicity.

Among UK Biobank participants there was highest adherence to the recommendations on physical activity and intake of sugar-sweetened drinks, and the sub-recommendation to eat at least 400 g of a variety of non-starchy vegetables and fruits every day. The recommendations with lowest adherence were those for the intake of red and processed meat and of alcoholic drinks. In addition, only 15% of participants reached the 30 g/day recommended intake for the dietary fibre sub-recommendation.

Participants with higher qualifications equivalent to an ISCED of 5 (College or University degree or A/AS levels or equivalent) had higher adherence scores compared with those with lower education qualifications (ISCED < 5). Total adherence scores for male participants were 12% lower than those for female participants, and male participants had higher BMIs, higher intakes of processed meats and alcohol and consumed less fruits and vegetables.

Since health-related behavioural patterns tend to cluster,^30^ as expected, we observed statistically significant, although weak, correlations between scores for individual recommendations such as ‘be a healthy body weight’ and ‘be physically active’. Surprisingly, we found inverse, but weak, correlations between scores for the recommendation to limit alcohol intake and ultra-processed food consumption, sugar-sweetened drinks intake and physical activity. The observed relationships in our analysis were not linked to differences in other sociodemographic factors such as socioeconomic status and education level and these findings should be explored further in future analyses.

### What is already known on this topic

While a recent study by Karavasiglou *et al*.^31^ assessed adherence to the Cancer Prevention Recommendations in the UK Biobank, this included female participants only and did not apply the standardized scoring system fully.^7^ These authors constructed a modified scoring system (‘lifestyle score’) based on six components.^31^ An earlier study also included only female participants in UK Biobank and created a modified ‘healthy lifestyle index’ based on five of the eight score components.^32^ In the CALIPER UK study, we have strengthened estimates of adherence by combining dietary data collected from the touchscreen questionnaire with those from the 24-hour dietary assessments, and using anthropometric and physical activity data, to fully operationalize the standardized scoring system for both males and females.^25^ In keeping with the aim of the ‘2018 WCRF/AICR Score’, this will allow robust comparison of our findings with those from other studies and populations.

The observed differences between men and women are consistent with a global comparison of diet quality which showed that, on average, women had better diets than men.^33^ In an earlier examination of diet quality—assessed as adherence to UK dietary recommendations for macronutrients, using data from UK Biobank—Bennett *et al*. ^22^ reported that men had greater non-adherence for total carbohydrates, polyunsaturated fat and protein whereas women had greater non-adherence for total sugar, fibre, total fat and saturated fat. Our findings that total adherence scores were lower in White and in Black participants compared with other ethnic groups, and in younger participants (aged ≤ 57), are in line with those by Mutz *et al.*  ^34^ who reported that UK Biobank participants of non-white ethnic background had higher odds of being healthy.

Our findings for inverse associations between scores for alcohol intake and physical activity are in line with those by Powell *et al.*^35^ who reported a greater proportion of physically active individuals, and lower proportion of inactive individuals, for participants with higher alcohol consumption. Furthermore, there was evidence that higher levels of physical activity attenuated the association between alcohol consumption and the risk of all-cause and cardiovascular disease mortality.^35^

### What this study adds

The mean dietary fibre intake across all participants was 22 g/day, which is similar to the estimated average fibre intake for adults in the UK (~20 g/day) and emphasizes the need for effective public health strategies to increase fibre intake.^36^ The mean values of individual score components used to assess adherence to the Cancer Prevention Recommendations differed significantly according to tertiles of total score and those with higher overall scores had higher adherence for every score component. From a public health perspective, this suggests that, in general, people in the UK with poorer adherence to the Cancer Prevention Recommendations will benefit from interventions that promote a healthier lifestyle overall, including diet, body weight and physical activity.

We have described the adherence of UK Biobank participants to the WCRF/AICR Cancer Prevention Recommendations, assessed using a standardized scoring system which allows for comparison with other studies and populations. We have shown that total adherence scores, and adherence to individual recommendations, are associated with sociodemographic factors such as ethnicity, area-based deprivation, and education. Identifying and understanding lifestyle and dietary patterns according to such sociodemographic factors could help to guide public health strategies for the prevention of lifestyle-related cancers and other NCDs e.g. type 2 diabetes and cardiovascular disease.

### Limitations of this study

The characteristics of those participants in UK Biobank for whom we derived a ‘total score’ and, therefore, were included in our analysis were broadly similar in respect of sex, ethnicity and smoking status to the rest of the UK Biobank participants. However, participants in the current analysis were slightly less deprived and more had a college or university degree than the rest of the UK Biobank cohort. This may limit generalizability of our findings to the whole UK Biobank cohort but we included all participants for whom relevant dietary, physical activity and anthropometric data were available. It should be noted that participants in the UK Biobank study were more likely to be older, female and less socioeconomically deprived than the rest of the UK population who were eligible for recruitment to UK Biobank.^37^ In addition, UK Biobank participants were less likely to be obese, drank less alcohol and were less likely to be smokers, compared with the general population.^37^ This is more of a concern for external generalizability rather than for internal validity of our findings, and the implications are 2-fold: firstly, it suggests that the mean adherence scores here are likely to overestimate adherence in the UK population as a whole; and secondly, that future analyses of associations between adherence and NCDs in UK Biobank should be adjusted for socio-demographic factors.

A limitation of this study is that the dietary intake data were self-reported and may be prone to misreporting or recall bias. In addition, we used a combination of two dietary assessment methods to operationalize the diet-related components of the 2018 WCRF Score, which were collected at different timepoints.^25^ Bradbury *et al*.^28^ reported good agreement between dietary data collected using both methods, as well as good reproducibility between habitual diet estimates from responses to the touchscreen questionnaire at baseline and those collected four years later at a repeat visit which suggests that dietary intake patterns for these middle-aged participants was relatively stable.

## Supplementary Material

supplementary_material_revision_fdad218

## Data Availability

Data are available upon request from UK Biobank (www.ukbiobank.ac.uk).

## Notes

**Fiona C. Malcomson**, Lecturer in Human Nutrition

**Solange Parra-Soto**, PhD Student

**Liya Lu**, Research Associate

**Frederick Ho**, Senior Lecturer

**Carlos Celis-Morales**, Research Fellow

**Linda Sharp**, Professor of Cancer Epidemiology

**John C. Mathers**, Professor of Human Nutrition
