# Supplementary material for: Socio-demographic variation in adherence to the World Cancer Research Fund (WCRF)/American Institute for Cancer Research (AICR) Cancer Prevention Recommendations within the UK Biobank prospective cohort study
Source: J Public Health (Oxf). 2023 Nov 20;46(1):61–71. doi: 10.1093/pubmed/fdad218 (PMC10901269; doi:10.1093/pubmed/fdad218)
Supplement: supplementary_material_revision_fdad218 [file supplementary_material_revision_fdad218.docx]

**SUPPLEMENTARY MATERIAL**

**Supplementary Methods:**

**Operationalisation of a standardised scoring system to assess adherence to the WCRF/AICR Cancer Prevention Recommendations**

We assessed adherence to the 2018 WCRF/AICR Cancer Prevention Recommendations using the standardised scoring system (the ‘2018 WCRF/AICR Score’) created by Shams-White and colleagues(8) and UK Biobank data (Supplementary Table 2). Further details of the UK Biobank data used, and methodology applied to derive adherence scores, are described in Supplementary Material and in detail in Malcomson *et al.*(26).

Adherence to the ‘healthy weight’ recommendation was based on data on BMI and waist circumference. BMI was calculated from weight data, measured by trained research staff to the nearest 0.1kg using the Tanita BC-418 MA body composition analyser, and height data, measured using a Seca 202 height measure. Waist circumference, also assessed by research staff, was measured at the natural indent (or umbilicus if the natural indent could not be located) using a Seca 200 tape measure.

Adherence to the ‘physical activity’ recommendation was assessed using self-reported data on time spent in moderate to vigorous physical activity (MVPA) collected using a validated short form of the International Physical Activity Questionnaire (IPAQ)(30), which asked participants about the frequency, intensity and duration of walking, moderate-intensity and vigorous-intensity physical activity during last month.

For the five diet-based score components, data from the 24-hour dietary assessment were used to measure adherence to the following recommendations: to ‘Wholegrains, vegetables, fruits and beans’, “Fast-foods”’, and ‘Sugar-sweetened drinks’. Data from the touchscreen questionnaire, which comprised a short food frequency questionnaire including 29 questions on diet and 18 on alcohol capturing information on the frequency of consumption of major food groups, including fruits and vegetables and meat, in the last year, were used to assess adherence to ‘Red and processed meat’ and ‘Alcohol consumption’ recommendations. Because there is no consensus on appropriate cut-off, points for the “fast-foods” recommendation were based on dividing participants into tertiles according to ultra-processed food (UPF) intake as a percentage of total energy intake.

Participants were allocated 1 point for fully meeting, 0.5 points for partially meeting or 0 points for not meeting each recommendation (component of the score), using cut-offs defined in the standardised scoring system. For recommendations with two score sub-components (‘healthy weight’ and ‘wholegrains, vegetables, fruits and beans’), participants scored 0.5 points for fully meeting, 0.25 points for partially meeting or 0 points for not meeting each sub-component. Thus, score components were given an equal weighting and the maximum score for each component was 1 point. The eighth, and optional, ‘Breastfeeding’ component of the score was not operationalised due to lack of data. Scores for individual components and sub-components of the score were summed to yield a single score for each individual ranging from 0 to 7 points.

**Supplementary Table 1: Abbreviated descriptions of the World Cancer Research Fund/American Institute for Cancer Research 2018 Cancer Prevention Recommendations**

| **Cancer Prevention Recommendation** | **Abbreviation** |
| --- | --- |
| 1. Be a healthy weight | 1. Healthy weight 2. BMI 3. Waist circumference |
| 1. Be physically active | 2. Physical activity |
| 1. Eat a diet rich in wholegrains, vegetables, fruit, and beans | 3. Wholegrains, Vegetables, Fruit, and Beans  a. Fruits and vegetables  b. Dietary fiber |
| 1. Limit consumption of “fast foods” and other processed foods high in fat, starches or sugars | 4. “Fast-foods” |
| 1. Limit consumption of red and processed meat | 5. Red and processed meat |
| 1. Limit consumption of sugar-sweetened drinks | 6. Sugar-sweetened drinks |
| 1. Limit alcohol consumption | 7. Alcohol consumption |
| 1. For mothers: breastfeed your baby, if you can (optional) | 8. Breastfeeding |

**Supplementary Table 2: Standardised scoring system used to assess adherence to the 2018 WCRF/AICR Cancer Prevention Recommendations, as devised by Shams-White et al. (2019)(8)**

| **2018 WCRF/AICR Recommendation** | **Operationalization of Recommendations** | **Points** |
| --- | --- | --- |
| 1. Be a healthy weight | **BMI (kg/m^2^)** |  |
|  | 18.5–24.9 | 0.5 |
|  | 25–29.9 | 0.25 |
|  | <18.5 or ≥30 | 0 |
|  | **Waist circumference (cm (in))** |  |
|  | Men: <94 (<37) Women: <80 (<31.5) | 0.5 |
|  | Men: 94–<102 (37–<40) Women: 80–<88 (31.5–<35) | 0.25 |
|  | Men: ≥102 (≥40) Women: ≥88 (≥35) | 0 |
| 1. Be physically active | **Total moderate-vigorous physical activity (MET min/wk)** |  |
|  | ≥600 | 1 |
|  | 300–<600 | 0.5 |
|  | <300 | 0 |
| 1. Eat a diet rich in wholegrains, vegetables, fruit and beans | **Fruits and vegetables (g/day)** |  |
|  | ≥400 | 0.5 |
|  | 200–<400 | 0.25 |
|  | <200 | 0 |
|  | **Total fibre (g/day) (AOAC definition)** |  |
|  | ≥30 | 0.5 |
|  | 15–<30 | 0.25 |
|  | <15 | 0 |
| 1. Limit consumption of “fast foods” and other processed foods high in fat, starches or sugars | **Percent of total kcal from ultra-processed foods (aUPFs)** |  |
|  | Tertile 1 (lowest) | 1 |
|  | Tertile 2 | 0.5 |
|  | Tertile 3 (highest) | 0 |
| 1. Limit consumption of red and processed meat | **Total red meat and processed meat (g/wk)** |  |
|  | Red meat ≤500 and processed meat <21 | 1 |
|  | Red meat ≤500 and processed meat 21–<100 | 0.5 |
|  | Red meat >500 or processed meat ≥100 | 0 |
| 1. Limit consumption of sugar-sweetened drinks | **Total sugar-sweetened drinks (g/day):** |  |
|  | 0 | 1 |
|  | >0–≤250 | 0.5 |
|  | >250 | 0 |
| 1. Limit alcohol consumption | **Total ethanol (UK guidelines) (units/week)):** |  |
|  | 0 | 1 |
|  | ≤14 | 0.5 |
|  | >14 | 0 |

**Supplementary Table 3: UK Biobank self-reported highest qualification and equivalent International Standard for Classification of Education (ISCED) codes(40)**

| **Qualification** | **ISCED** |
| --- | --- |
| College or University degree | 5 |
| NVQ or HND or HNC or equivalent | 5 |
| Other professional qualification e.g. nursing, teaching | 4 |
| A levels/AS levels or equivalent | 3 |
| O levels/GCSEs or equivalent | 2 |
| CSEs or equivalent | 2 |
| None of the above | 1 |

A levels: Advanced levels, AS levels: Advance Subsidiary levels, CSE: Certificate of Secondary Education, GCSE: General Certificate of Secondary Education, HNC: Higher National Certificate, HND: Higher National Diploma, NVQ: National Vocational Qualifications, O levels: General Certificate of Education Ordinary Level

**Supplementary Table 4: Sociodemographic characteristics of UK Biobank participants with a total adherence score, who are included in the present analysis, and those without a total score, who were excluded**

|  | **Participants with a total score included in the present analysis** | **Participants without a total score excluded from this analysis** |
| --- | --- | --- |
| n | 158,415 | 344,121 |
| Females (%) | 84,463 (53.3) | 188,928 (54.9) |
| Men (%) | 73,952 (46.7) | 155,176 (45.1) |
| Age (years) | 56.1 (8.0) | 56.7 (8.2) |
| Townsend deprivation index | -1.63 (2.8) | -1.14 (3.2) |
| Ethnicity (%) |  |  |
| White | 151,646 (95.7) | 321,017 (93.3) |
| Mixed | 1,988 (1.3) | 5,528 (1.6) |
| South Asian | 2,175 (1.4) | 7,707 (2.2) |
| Black | 1,743 (1.1) | 6,318 (1.8) |
| Chinese | 433 (0.3) | 1,141 (0.3) |
| Smoking (%) |  |  |
| Never | 90,365 (57.0) | 183,147 (53.2) |
| Former | 56,092 (35.4) | 116,958 (34.0) |
| Current | 11,691 (7.4) | 41,286 (12.0) |
| Education (%) |  |  |
| College or university degree | 77,903 (49.2) | 109,054 (31.7) |
| A levels/AS levels or equivalent | 20,832 (13.2) | 34,488 (10.0) |
| O levels/GCSEs or equivalent | 32,047 (20.2) | 73,139 (21.3) |
| SEs or equivalent/NVQ or HND or HNC | 14,896 (9.4) | 44,714 (13.0) |

Data are presented as means and standard deviation (SD) or ‘n’ and percentage (%).

Data missing for subset of participants with a total score: 0.1% Townsend Deprivation Index, 0.3% ethnicity, 0.2% smoking status, 8% education.

Data missing for participants without score: 0.02% age, 0.1% Townsend Deprivation Index, 0.7% ethnicity, 0.8% smoking status, 24% education.

A levels: Advanced levels, AS levels: Advance Subsidiary levels, CSE: Certificate of Secondary Education, GCSE: General Certificate of Secondary Education, HNC: Higher National Certificate, HND: Higher National Diploma, NVQ: National Vocational Qualifications, O levels: General Certificate of Education Ordinary Level

**Supplementary Table 5. Mean values for components of the total score for adherence to the Cancer Prevention Recommendations according to tertiles of total score**

| **Score components** | **Lowest**  **(0 – 3.5)** | **Medium**  **(3.75 – 4.25)** | **Highest**  **(4.5 – 7)** | **P value** |
| --- | --- | --- | --- | --- |
| **Total score** | 2.84 (0.60) | 4.00 (0.20) | 5.02 (0.49) | **<0.001** |
| **1. Healthy weight**  **a. BMI (kg/m^2^)** | 28.7 (4.8) | 26.5 (4.1) | 24.7 (3.5) | **<0.001** |
| **b. Waist circumference (cm)**  Females  Males | 89.0 (12.6)  99.7 (11.1) | 83.0 (11.3)  93.6 (9.6) | 78.0 (9.5)  89.5 (8.5) | **<0.001**  **<0.001** |
| **2. Physical activity (MVPA in MET mins/wk)** | 950 (1601) | 1671 (1873) | 2146 (1968) | **<0.001** |
| **3a. Fruit and vegetable intake (g/d)** | 302 (212) | 415 (235) | 546 (279) | **<0.001** |
| **b. Dietary fiber (g/d)^a^** | 19.6 (7.5) | 21.9 (7.8) | 25.1 (9.1) | **<0.001** |
| **4. “Fast-foods” (UPF intake as % of total energy intake/d)** | 65.8 (181.2) | 54.7 (146.3) | 40.0 (140.9) | **<0.001** |
| **5. Red meat intake (g/week)** | 280 (176) | 251 (159) | 200 (146) | **<0.001** |
| **Processed meat intake (g/week)** | 103.3 (78.2) | 71.5 (67.4) | 42.0 (49.9) | **<0.001** |
| **7. Sugar-sweetened drinks (drinks/d)** | 0.61 (0.88) | 0.36 (0.66) | 0.21 (0.51) | **<0.001** |
| **8. Alcohol consumption (units/week)** | 18.4 (14.3) | 15.4 (12.4) | 12.6 (10.1) | **<0.001** |

Data are presented as mean (standard deviation) or n (%). Differences according to score tertiles were analyses using One-Way ANOVA.

^a^Dietary fibre assessed using Association of Analytical Chemists (AOAC) method, MVPA: moderate-vigorous physical activity, UPF: ultra-processed foods.
